# Association between the Big Five personality traits and medication adherence in patients with cardiovascular disease: A cross-sectional study

**DOI:** 10.1371/journal.pone.0278534

**Published:** 2022-12-01

**Authors:** Takuji Adachi, Yuki Tsunekawa, Daisuke Tanimura

**Affiliations:** 1 Department of Integrated Health Sciences, Nagoya University Graduate School of Medicine, Nagoya, Japan; 2 Department of Rehabilitation, Nagoya Ekisaikai Hospital, Nagoya, Japan; 3 Department of Cardiology, Nagoya Ekisaikai Hospital, Nagoya, Japan; Saud Al-Babtain Cardiac Centre, SAUDI ARABIA

## Abstract

The correlation between personality traits and health outcomes of primary prevention has been examined. However, there is a lack of evidence on the association between the assessment of personality traits and medication adherence for secondary prevention of cardiovascular disease. Thus, this study aimed to explore the association between personality traits and medication adherence, including compliance to prescribed medications and attitudes toward taking medications among patients with cardiovascular disease. This cross-sectional study included patients hospitalized for cardiovascular disease. We assessed the Big Five personality traits (conscientiousness, neuroticism, openness, extraversion, and agreeableness) of each patient at discharge using the Ten-Item Personality Inventory. In addition, we evaluated four aspects of medication adherence using a 12-item version of the medication adherence scale: medication compliance, collaboration with health care providers, willingness to access and use information on medication, and acceptance to take medication. Logistic regression analysis was performed to assess the correlation between the level of each medication adherence domain and each personality trait. The data of 128 patients with cardiovascular disease were analyzed. Higher conscientiousness score was significantly associated with a high compliance score (odds ratio per 1 point increase, 1.90; 95% confidence interval, 1.30–2.79; p = 0.001), high collaboration score (1.90; 1.31–2.76; p = 0.001), and high willingness score (1.74; 1.19–2.54; p = 0.004) after adjustment for potential confounders. Other combinations of personality traits and medication adherence showed no statistically significant correlations in multivariate analyses. The findings of this study suggest that assessment of personality traits, especially conscientiousness, may facilitate patient–medical staff communication for the improvement of medication adherence in patients with cardiovascular disease.

## Introduction

Medication adherence is a key factor in the secondary prevention of cardiovascular disease (CVD) [1]. Several cardioprotective drugs have been established as first-line treatment for heart failure, especially among patients with reduced ventricular ejection fraction [2]. In addition, optimal medical therapy is increasingly being considered the cornerstone of treatment for patients with acute and chronic coronary syndrome [3]. Furthermore, the guidelines for the prevention of CVD indicate that controlling recognized cardiovascular risk factors is a requirement for safe and effective cardiac rehabilitation [4]. Thus, guideline-directed medical therapy is crucial for lowering the risk of mortality and recurrence in patients with various CVDs. However, a meta-analysis demonstrated that the prevalence of good compliance to taking cardiovascular medications as prescribed ranges from 50% to 70% and that 9% of all cardiovascular events may be attributed to poor medication compliance [5]. Recent studies have focused on multiple aspects of medication “adherence,” a concept that includes patients’ understanding of medications and active participation in treatment decisions [6]. According to a recent systematic review, medication adherence scales with multidomain have been increasingly used in studies as patient-reported outcome measures [7]. Therefore, a multidisciplinary behavioral approach and effective patient–medical staff communication for the improvement of medication adherence, including patients’ attitude toward taking medications, play key roles in secondary prevention of CVD.

Personality traits have been proposed as fundamental constructs of health-related behavior, and communication with health care providers [8] may also help in understanding the interests or concerns of patients regarding cardiovascular therapy. The Big Five model, also known as the five-factor model, is a widely accepted model of personality traits [9, 10], which are closely associated with the long-term health outcomes of primary prevention of CVD [11]. As the name suggests, the Big Five model includes five personality traits: conscientiousness, neuroticism, openness, extraversion, and agreeableness. Conscientiousness reflects the propensity to follow rules and be self-controlled, task- and goal-directed, and planful. Neuroticism contrasts even-temperedness with the experience of anxiety, worry, anger, and depression. Openness refers to the proneness to be original, complex, creative, and open to new ideas. Extraversion refers to the propensity to be sociable, active, and assertive and to have a positive affect. Agreeableness refers to the degree to which a person needs pleasant and harmonious relationships with others [9, 10]. Since beliefs on medicine can influence patients’ decisions for taking medications [12], several studies have attempted to explore the relationship between personality traits and medication adherence. According to these studies, conscientiousness and neuroticism are associated with adherence to medications and doctor’s regimens [13, 14]. In addition, a recent study among patients with CVD reported an association between low conscientiousness and high dropout rate of cardiac rehabilitation [15]. Regarding lifestyle behaviors, low conscientiousness and high neuroticism are associated with unfavorable lifestyle habits such as physical inactivity, smoking, alcohol consumption, and unhealthy eating [16–18]. Therefore, assessment of personality traits may provide insights, which can help facilitate effective communication for medication adherence among patients with CVD.

The correlation between personality traits and health outcomes of primary prevention has been examined. However, there is a lack of evidence on the association between assessment of personality traits and medication adherence for secondary CVD prevention. Therefore, this study aimed to explore the association between the Big Five personality traits and medication adherence in patients with CVD.

## Methods

### Study design and participants

In this cross-sectional study, we retrospectively retrieved and analyzed the data of patients with CVD admitted at Nagoya Ekisaikai Hospital in Nagoya City, Japan, between April 2021 and March 2022. The inclusion criteria were participation in an inpatient cardiac rehabilitation program and completion of a routine clinical assessment questionnaire during hospitalization. Patients with one or more of the following conditions were excluded: not receiving prescribed medications for chronic disease before admission, inability to walk, unable to fill in the questionnaire owing to visual or hearing impairment, presence of severe psychiatric or neurological disorders, presence of physician-diagnosed dementia or taking anti-dementia drugs (N06D in the Anatomical Therapeutic Chemical Classification System) before admission.

In Japan, inpatient cardiac rehabilitation has become standard care for patients hospitalized for CVD. In 2017, the implementation rates for inpatient cardiac rehabilitation were 76.5%, 65.6%, and 46.9% for patients hospitalized for cardiac surgery, acute coronary syndrome, and heart failure, respectively [19], which constitute the main population of this study. Health insurance is mandatory in Japan, and cardiac rehabilitation is covered by the Japanese health care system. Hence, the participants in the present study did not require special medical care. In inpatient cardiac rehabilitation, all patients received rehabilitation based on the Japanese Circulation Society guidelines [20]. Pharmacists and nurses provided each patient with guidance for taking prescribed medication as routine patient care during hospitalization.

### Personality traits

The personality traits of each patient were assessed during routine clinical practice using the Japanese version of the Ten-Item Personality Inventory (TIPI-J) [21]. The original Ten-Item Personality Inventory scale is widely used as a brief scale for the evaluation of the Big Five personality traits [22]. The TIPI-J includes two items on each of the Big Five domains, making a total of 10 items. Each domain is assessed using one positively keyed item and one negatively keyed item. The responses for each item are graded using a seven-category response scale, and the average score of the two items included in each trait are calculated to determine the score for each trait (ranging from 1 to 7 points). Higher scores indicate a higher level of the trait. The reliability and validity of the TIPI-J have been reported [21]. In addition, the reliability and concurrent validity of the TIPI-J for the assessment of older individuals have been confirmed [23].

### Medication adherence

The World Health Organization has highlighted the need for patient consent to and participation in treatment [6], suggesting that health communication between patients and health care providers is an important psychosocial aspect of medication support and treatment decision making. This concept is consistent with several reports on medication adherence among people with chronic diseases [24, 25]. Therefore, we evaluated multiple aspects of medication adherence in the present study using a 12-item version of the medication adherence scale developed by Ueno et al. [26] This scale includes four subscales on adherence: medication compliance (e.g., “I have stopped taking medication based on my own judgment [not including times when I forgot to take my medication]”); collaboration with health care providers (e.g., “I feel comfortable asking my health care provider about my medication”); willingness to access and use information on medication (e.g., “I understand both the effects and the side effects of my medication”); and acceptance to take medication and how taking medication fits the patient’s lifestyle (e.g., “I accept the necessity of taking medication in the prescribed manner to treat my illness”). The items for medication compliance were answered based on pre-hospitalization experience, and the other items were answered based on perspective and attitudes toward the treatment and medications at the time of response. Each subscale contains three items that are answered using a five-category response scale. The sum of the scores of the three items for each subscale ranges from 3 to 15 points. Higher scores indicate higher medication adherence. The reliability and validity of the scale for the assessment of patients with chronic diseases, including heart disease and coronary risk factors, have been confirmed [26]. In addition, the usefulness of this medication adherence scale for the assessment of elderly adults has been examined [27].

### Clinical data

Patients’ medical records were reviewed to extract data on age, sex, body mass index, principal etiology, comorbidities, left ventricular ejection fraction, biochemical parameters, medications prescribed at discharge, need for a walking device, or need for walking assistance during hospital stay. Comorbidities were evaluated using the Charlson Comorbidity Index [28].

### Statistical analysis

Continuous variables are expressed as mean and standard deviation (SD) for normally distributed variables and as median with interquartile range for non-normally distributed data. Categorical data are expressed as numbers and percentages.

Some subscale scores of medication adherence showed skewed distributions. Therefore, each subscale was categorized into binary variables of high and low levels using median values. Thereafter, logistic regression analysis was performed to evaluate the relationship between medication adherence (high/low) and the Big Five personality traits. To explore their potential correlations, logistic regression analysis adjusted for age was first performed, with medication adherence as a dependent variable and personality trait as an independent variable. Thereafter, multivariate analyses were further performed adjusted for the potential confounders if the relationships were significant in the age-adjusted analyses. The number of independent variables was based on the concept of one variable per 10 outcome events in the logistic regression analysis [29]. To follow this concept as closely as possible, a logistic regression model that included six variables was used in multivariate analyses.

All statistical analyses were performed using Stata/SE software version 15.1 (StataCorp LP). A p-value of <0.05 was considered statistically significant. This study was an exploratory analysis of the association between personality traits and multidimensional medication adherence; thus, no adjustment for multiple testing was performed. Considering the potential for type I error, this study should be considered a hypothesis-generating study.

### Ethical considerations

This study was performed according to the principles of the Declaration of Helsinki and was approved by the ethics committee of Nagoya Ekisaikai Hospital (Approval No.: 2019–043). The requirement to obtain informed consent from the patients was waived because of the retrospective nature of the study. Instead, all patients were informed about their participation in this study and each patient was offered the opportunity to opt out of the study. Information regarding this study, such as the inclusion criteria and opportunity to opt out, was provided on the hospital’s website. The ethics committees approved this consent procedure. No patient opted out of the study at the time of analysis.

## Results

**Fig 1:**
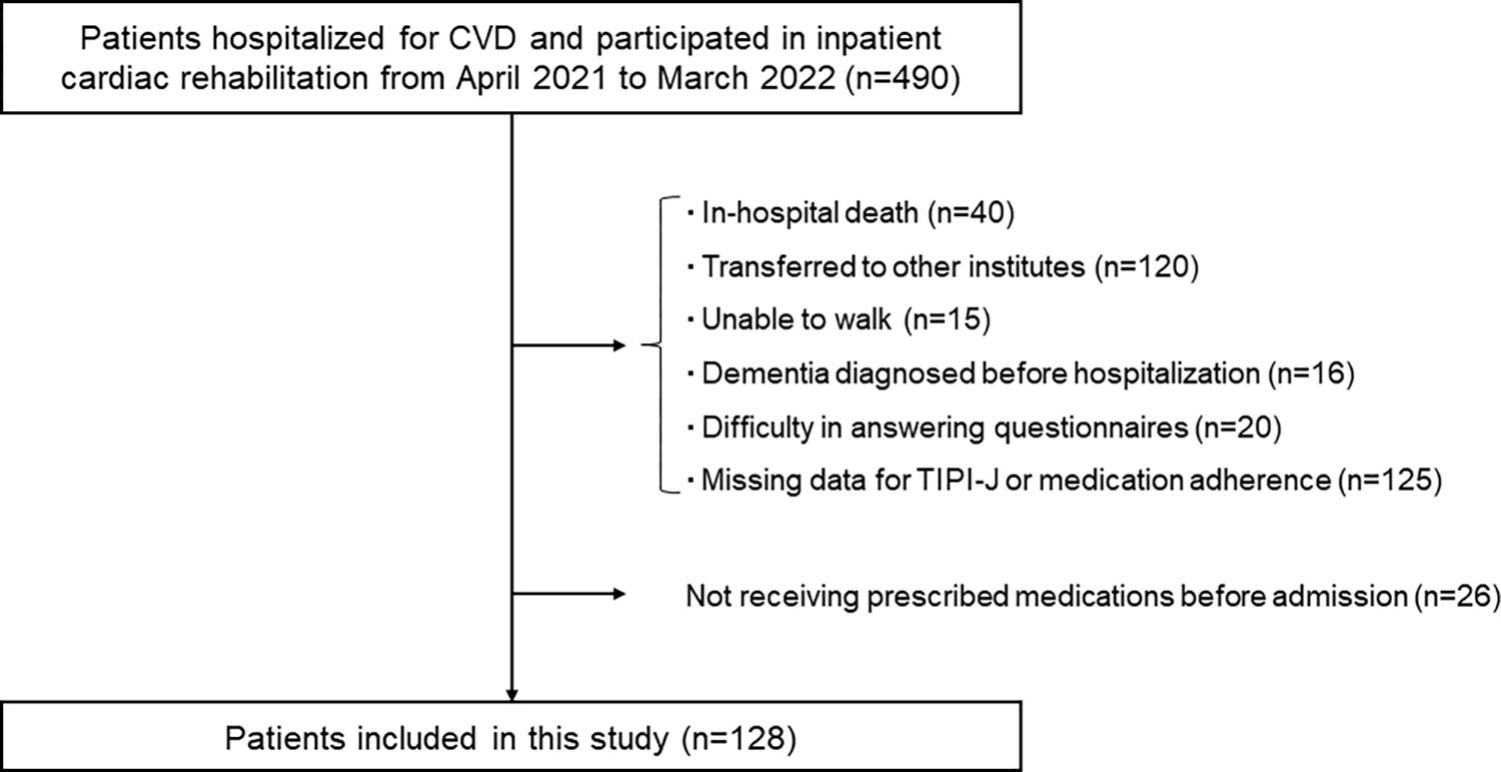
Flowchart of the study participants. CVD, cardiovascular disease; TIPI-J, Japanese version of the Ten-Item Personality Inventory.

A total of 128 patients hospitalized for CVD were analyzed (Fig 1). The median age of the patients was 70 years (interquartile range, 58–78 years), and 73.4% patients were male. The mean body mass index of the patients was 22.9 kg/m^2^ (SD, 4.3 kg/m^2^), and 41.4% patients were hospitalized due to acute coronary syndrome (Table 1). The distributions of the Big Five personality traits and medication adherence scores are summarized in Table 2. Comparisons of personality traits according to several patient characteristics are presented in S1 Table. Patients with older age (≥70 years) had lower neuroticism and agreeableness levels compared to younger patients (<70 years). Patients with heart failure showed a lower agreeableness level compared to those without heart failure. There were no differences in personality traits according to sex and number of prescribed medications.

**Table 1:** Characteristics of the study participants (n = 128).

	Median (IQR), Mean (SD), or %
Age, years	70 (58–78)
Men	73.4%
Body mass index, kg/m^2^	22.9 (4.3)
Reason for hospitalization	
Acute coronary syndrome	41.4%
Heart failure	32.8%
Cardiac surgery	23.4%
Others	2.3%
Comorbidities	
Hypertension	58.6%
Dyslipidemia	40.6%
Diabetes mellitus	28.1%
Prior heart failure	14.8%
Stroke	9.4%
Charlson Comorbidity Index, points	1 (1–2)
LVEF, % (n = 118)	50 (44–61)
Prescribed medication	
Beta blocker	75.8%
ACEi/ARB	56.3%
MRA	35.2%
Diuretic	33.6%
Satin	78.1%
Antithrombotic agent	66.4%
Anticoagulant	65.6%
Number of prescribed medications	5 (4–7)
Length of hospital stay, days	16 (12–20)
Living alone	18.1%

IQR, interquartile range; SD, standard deviation; LVEF, left ventricular ejection fraction; ACEi, angiotensin converting enzyme inhibitor; ARB, angiotensin II receptor blocker; MRA, mineralocorticoid receptor antagonist

**Table 2:** Distribution of Big Five personality traits and medication adherence scores.

	Median (IQR)	Mean (SD)
Big Five personality traits		
Conscientiousness	4.0 (3.5–5.0)	4.10 (1.15)
Neuroticism	4.0 (3.5–4.5)	3.07 (1.00)
Openness	4.0 (3.0–4.5)	3.80 (1.08)
Extraversion	4.0 (3.5–4.5)	4.09 (1.11)
Agreeableness	3.5 (2.5–4.0)	3.20 (1.16)
Medication adherence		
Compliance score	10 (9–11)	9.72 (1.77)
Collaboration score	12 (10–12)	10.99 (2.38)
Willingness score	9 (8–11)	9.14 (2.62)
Acceptance score	11 (10–13)	11.31 (2.13)

IQR, interquartile range; SD, standard deviation

Each Big Five personality trait was normally distributed.

Among the medication adherence scores, compliance and acceptance were normally distributed, and collaboration and willingness showed right-skewed distribution.

**Fig 2:**
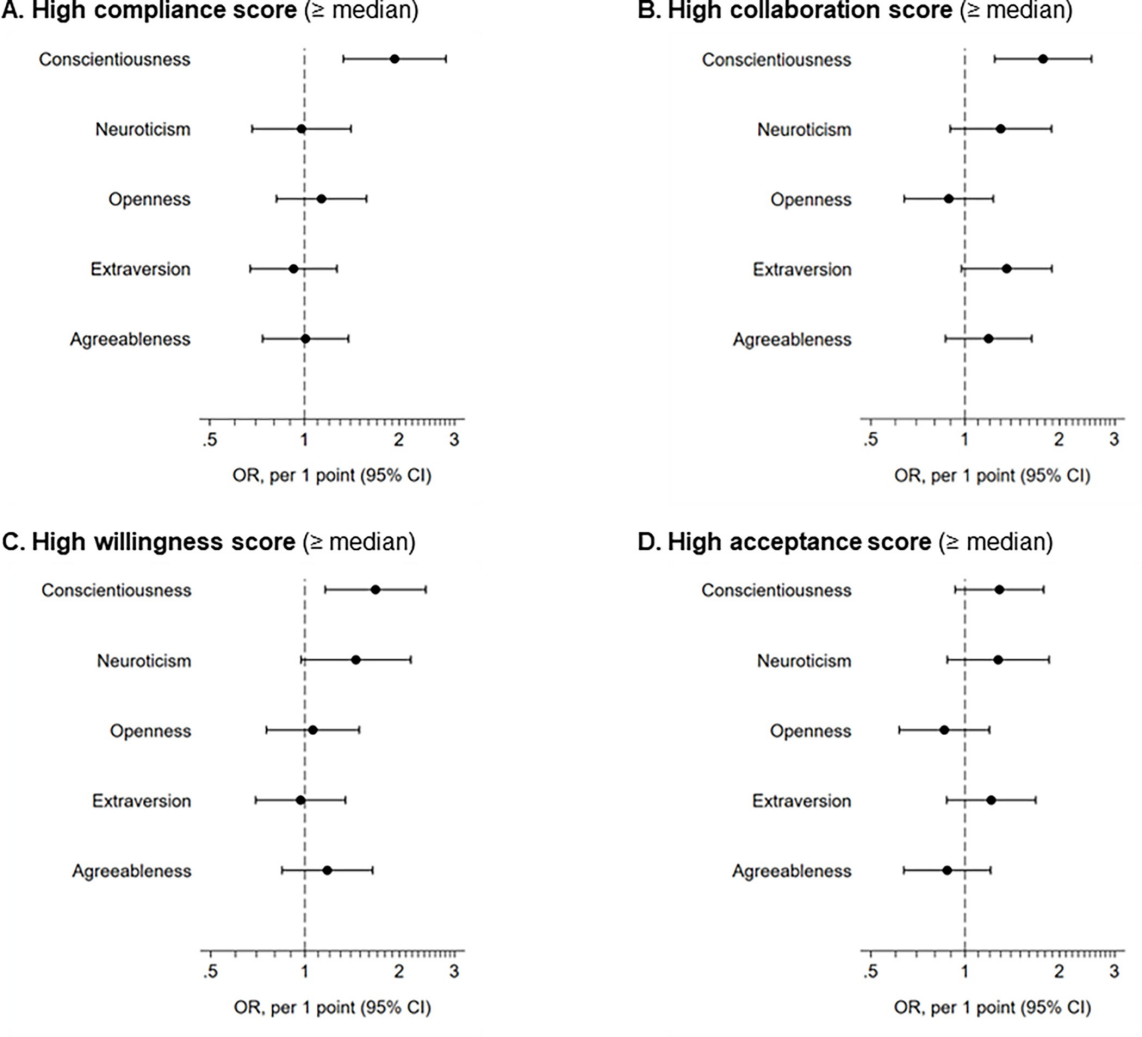
Results of age-adjusted logistic regression analysis. OR, odds ratio; CI, confidence interval.

Fig 2 shows the results of the logistic regression analysis adjusted for age. Higher conscientiousness was associated with high compliance, high collaboration, and high willingness scores (p<0.05). Higher neuroticism tended to be associated with high willingness score (p = 0.069). Higher extraversion tended to be associated with a high collaboration score; however, the result was not statistically significant (p = 0.071).

The results of the multivariate logistic regression analysis are presented in Table 3. After adjustment for age, sex, heart failure, number of medications prescribed, and Charlson Comorbidity Index, higher conscientiousness was significantly associated with a high compliance score (odds ratio per 1 point increase, 1.90; 95% confidence interval, 1.30–2.79; p = 0.001), high collaboration score (1.90; 1.31–2.76; p = 0.001), and high willingness score (1.74; 1.19–2.54; p = 0.004). The results of all multivariate analyses are summarized in S2 Table.

**Table 3:** Results of multivariate logistic regression analysis.

Dependent variable	Independent variables	OR	(95% CI)	p
High compliance	Conscientiousness, per 1 pt	1.90	(1.30–2.79)	0.001
	Age, per 1yr	1.00	(0.96–1.03)	0.819
	Sex (women)	0.79	(0.31–2.01)	0.621
	Complication of heart failure	1.01	(0.38–2.66)	0.983
	Number of prescribed medications, per 1 drug	0.93	(0.74–1.17)	0.549
	Charlson Comorbidity Index, per 1 pt	1.25	(0.76–2.07)	0.380
High collaboration	Conscientiousness, per 1 pt	1.90	(1.31–2.76)	0.001
	Age, per 1yr	0.97	(0.94–1.01)	0.110
	Sex (women)	0.78	(0.31–1.96)	0.603
	Complication of heart failure	0.83	(0.31–2.20)	0.701
	Number of prescribed medications, per 1 drug	1.21	(0.97–1.53)	0.091
	Charlson Comorbidity Index, per 1 pt	0.94	(0.58–1.54)	0.817
High willingness	Conscientiousness, per 1 pt	1.74	(1.19–2.54)	0.004
	Age, per 1yr	0.95	(0.92–0.98)	0.004
	Sex (women)	0.95	(0.38–2.36)	0.904
	Complication of heart failure	1.00	(0.36–2.76)	0.994
	Number of prescribed medications, per 1 drug	1.12	(0.89–1.42)	0.345
	Charlson Comorbidity Index, per 1 pt	0.91	(0.55–1.51)	0.718

OR, odds ratio; CI, confidence interval

Complication of heart failure is defined as heart failure hospitalization and/or prior heart failure.

## Discussion

In this cross-sectional study, we explored the association between multiple aspects of the Big Five personality traits and medication adherence in patients with CVD. The results indicated that a higher level of conscientiousness, a personality trait known to be related to a healthy lifestyle, was positively associated with medication compliance, collaboration with health care providers, and willingness to access and use medical information. Although this study should be considered a hypothesis-generating study due to the exploratory analysis performed during data analysis, the findings suggest that assessment of personality traits may provide clues for patient–medical staff communication in promoting medication adherence.

The positive association between high conscientiousness and favorable medication compliance observed in the present study corroborates the results of a previous meta-analysis of patients with chronic diseases, including hypertension and diabetes mellitus [13]. The results of the present study imply that conscientiousness is also a predictive factor for good compliance to taking prescribed medications for secondary prevention of CVD. Although data on the possible underlying mechanisms of the relationship between conscientiousness and medication compliance was not elucidated in the present study, low conscientiousness may reflect forgetfulness, a known characteristic of non-adherence [30]. The authors of a recent systematic review recommended the use of reminder tools for patients who forget to take prescribed medications [31]. Additionally, a previous study on patients with CVD demonstrated the correlation between conscientiousness and self-efficacy in following the recommendations of the medical team [32]. Therefore, medical professionals may need to consider tailoring medical communication according to a patient’s subjective conscientiousness and self-efficacy for more effective disease management.

Another novel finding of the present study is the potential relationship between conscientiousness and collaboration with health care providers and willingness to access medical information. To date, research on the association between personality traits and medication adherence has focused on compliance to taking medication as prescribed [33, 34]. However, there is a growing interest in the psychological aspect of medication adherence [6, 24, 25]. Therefore, we evaluated multiple aspects of medication adherence in the present study. The collaboration score assessed in this study reflects whether patients can communicate with medical staff. Meanwhile, the willingness score indicates the ability to collect medical information associated with prescribed medication and understand its effects and side effects. Since patients usually do not directly feel the effects of cardioprotective medications or drugs used for controlling CVD risk factors, understanding the reasons for taking prescribed medications is key for enhancing long-term adherence [30]. Indeed, several trials have been conducted with the aim of improving medication adherence by promoting patient understanding through education and counseling in addition to providing reminders [31]. However, further longitudinal research is needed to examine whether the collaboration and willingness aspects of medication adherence are related to future medication compliance and subsequent improved clinical outcomes in patients with CVD.

In the present study, extraversion tended to be associated with a high collaboration score, although they were not statistically significant. This result may support the findings reported by a previous study on patients with diabetes mellitus, which showed that extraversion was associated with medication adherence, especially among older individuals [34]. Since the average age of patients with CVD has increased in Japan [35], extraversion may be helpful for patients’ understanding or communication with health care providers in secondary CVD prevention. However, this personality trait had no correlation with compliance in taking prescribed medication. Since the medication compliance in this study reflected pre-hospitalization condition, further assessments need to be conducted on post-discharge disease management.

Neuroticism has a negative effect on medication adherence [33, 34]; however, such a relationship was not observed in the present study. In contrast to previous studies, the present study included hospitalized patients who had recently developed or have worsened CVDs. Therefore, patients’ apprehension on illness-specific symptoms of CVD could likely affect the results of the present study. Another study on patients with asthma reported that patients with negative affectivity tended to be more aware of and correctly report disease-specific symptoms [36]. Openness is another personality trait related to the use of complementary and alternative medicine or low medication adherence [37]; however, this was not observed on the present study. The presence of symptoms and subjective severity of disease may affect the association between personality traits and attitudes toward lifestyle and medical treatment.

Several studies on agreeableness and healthy lifestyle have reported varied results [11, 16, 34, 38, 39]. Although this trait refers to one’s tendency to prioritize interpersonal relationships [9, 10], it was not correlated with any domains of medication adherence in this study. This may be due to a risk of bias as this study is a single center retrospective analysis. Additionally, agreeableness was lower in older patients with heart failure, suggesting the necessity of subgroup analysis based on several patient characteristics. Because of the limited sample size of this study, further research on the correlation between agreeableness and health behaviors, especially with regard to secondary prevention of CVD, is needed.

This study has some limitations. First, medication adherence was assessed using a self-reported questionnaire. Of the domains of adherence assessed in this study, pill counts or electronic lids should be considered for compliance in taking prescribed medications. Second, there was a potential for selection bias in the present study due to its single-center retrospective design. Hence, the generalizability of our results should be carefully considered. Third, due to the limited size of the study population, not all confounding factors with available data were considered in multivariate analyses. Additionally, subgroup analysis for sensitivity analyses of different patient characteristics could not be performed owing to the small sample size of this study. Fourth, several potential confounding factors, such as genetic predisposition and socioeconomic status, were not assessed in this study. Therefore, as discussed previously, this exploratory analysis should be considered a hypothesis-generating study. Finally, as this was a cross-sectional analysis, the longitudinal association between personality traits and actual health behavior after discharge was not evaluated.

## Conclusions

This study shows that a high level of conscientiousness is positively associated with several aspects of medication adherence, including acceptance to take medications, collaboration with health care providers, and willingness to access and use medical information. Although this study should be considered a hypothesis-generating study due to the exploratory nature of the analysis, its findings suggest that assessment of personality traits may provide clues that facilitate patient–medical staff communication in promoting medication adherence.

## Supporting information

S1 Table Big Five personality traits according to the patient characteristics. (DOCX)

S2 Table Results of multivariate logistic regression analysis for the association between Big Five personality traits and four domains of medication adherence (dependent variable). (DOCX)

S1 Dataset (XLSX)

## References

[1] Visseren FLJ, MacH F, Smulders YM, Carballo D, Koskinas KC, Bäck M, et al. 2021 ESC Guidelines on cardiovascular disease prevention in clinical practice. Eur Heart J. 2021;42: 3227–3337. doi: 10.1093/eurheartj/ehab484 34458905

[2] McDonagh TA, Metra M, Adamo M, Gardner RS, Baumbach A, Böhm M, et al. 2021 ESC Guidelines for the diagnosis and treatment of acute and chronic heart failure. Eur Heart J. 2021;42: 3599–3726. doi: 10.1093/eurheartj/ehab368 34447992

[3] Brown DL. Optimal Medical Therapy as First-Line Therapy for Chronic Coronary Syndromes: Lessons from COURAGE, BARI 2D, FAME 2, and ISCHEMIA. Cardiovasc drugs Ther. 2021. doi: 10.1007/s10557-021-07289-6 34767134

[4] Ambrosetti M, Abreu A, Corrà U, Davos CH, Hansen D, Frederix I, et al. Secondary prevention through comprehensive cardiovascular rehabilitation: From knowledge to implementation. 2020 update. A position paper from the Secondary Prevention and Rehabilitation Section of the European Association of Preventive Cardiology. Eur J Prev Cardiol. 2021;28: 460–495. doi: 10.1177/2047487320913379 33611446

[5] Chowdhury R, Khan H, Heydon E, Shroufi A, Fahimi S, Moore C, et al. Adherence to cardiovascular therapy: A meta-analysis of prevalence and clinical consequences. Eur Heart J. 2013;34: 2940–2948. doi: 10.1093/eurheartj/eht295 23907142

[6] World Health Organization. Adherence to long-term therapies: Evidence for action. [cited 4 Jul 2022]. Available: http://apps.who.int/medicinedocs/en/d/Js4883e/1.html

[7] Tegegn HG, Wark S, Tursan d’Espaignet E, Spark MJ. Measurement Properties of Patient-Reported Outcome Measures for Medication Adherence in Cardiovascular Disease: A COSMIN Systematic Review. Clin Drug Investig. 2022. doi: 10.1007/s40261-022-01199-7 36180813 PMC9617955

[8] Ferguson E. Personality is of central concern to understand health: towards a theoretical model for health psychology. Health Psychol Rev. 2013;7: S32–S70. doi: 10.1080/17437199.2010.547985 23772230 PMC3678852

[9] Costa PT, McCrae RR. Personality in Adulthood: A Six-Year Longitudinal Study of Self-Reports and Spouse Ratings on the NEO Personality Inventory. J Pers Soc Psychol. 1988;54: 853–863. doi: 10.1037//0022-3514.54.5.853 3379583

[10] McCrae RR, John OP. An Introduction to the Five‐Factor Model and Its Applications. J Pers. 1992;60: 175–215. doi: 10.1111/j.1467-6494.1992.tb00970.x 1635039

[11] Jokela M, Pulkki-Råback L, Elovainio M, Kivimäki M. Personality traits as risk factors for stroke and coronary heart disease mortality: pooled analysis of three cohort studies. J Behav Med. 2014;37: 881–889. doi: 10.1007/s10865-013-9548-z 24203126

[12] Horne R, Weinman J. Patients’ beliefs about prescribed medicines and their role in adherence to treatment in chronic physical illness. J Psychosom Res. 1999;47: 555–567. doi: 10.1016/s0022-3999(99)00057-4 10661603

[13] Molloy GJ O’Carroll RE, Ferguson E. Conscientiousness and medication adherence: A meta-analysis. Ann Behav Med. 2014;47: 92–101. doi: 10.1007/s12160-013-9524-4 23783830

[14] Wheeler K, Wagaman A, McCord D. Personality traits as predictors of adherence in adolescents with type I diabetes. J Child Adolesc Psychiatr Nurs. 2012;25: 66–74. doi: 10.1111/j.1744-6171.2012.00329.x 22512523

[15] Adachi T, Tsunekawa Y, Matsuoka A, Tanimura D. Association between Big Five Personality Traits and Participation in Cardiac Rehabilitation in Japanese Patients with Cardiovascular Disease: A Retrospective Cohort Study. Int J Environ Res Public Health. 2021;18: 8589. doi: 10.3390/ijerph18168589 34444339 PMC8392722

[16] Sutin AR, Stephan Y, Luchetti M, Artese A, Oshio A, Terracciano A. The five-factor model of personality and physical inactivity: A meta-analysis of 16 samples. J Res Pers. 2016;63: 22–28. doi: 10.1016/j.jrp.2016.05.001 29056783 PMC5650243

[17] Mroczek DK, Spiro A, Turiano NA. Do health behaviors explain the effect of neuroticism on mortality? Longitudinal findings from the VA Normative Aging Study. J Res Pers. 2009;43: 653–659. doi: 10.1016/j.jrp.2009.03.016 20161240 PMC2705907

[18] Bogg T, Roberts BW. Conscientiousness and health-related behaviors: A meta-analysis of the leading behavioral contributors to mortality. Psychol Bull. 2004;130: 887–919. doi: 10.1037/0033-2909.130.6.887 15535742

[19] Kanazawa N, Yamada S, Fushimi K. Trends in the Use of Cardiac Rehabilitation in Japan Between 2010 and 2017 ― An Epidemiological Survey ―. Circ Reports. 2021;3: 569–577. doi: 10.1253/circrep.cr-21-0018 34703934 PMC8492403

[20] Japanese Circulation Society. JCS/JACR 2021 Guideline on Rehabilitation in Patients with Cardiovascular Disease. 2021 [cited 14 Jul 2022]. Available: https://www.j-circ.or.jp/cms/wp-content/uploads/2021/03/JCS2021_Makita.pdf 10.1253/circj.CJ-22-0234 36503954

[21] Oshio A, Abe S, Cutrone P. Development, Reliability, and Validity of the Japanese Version of Ten Item Personality Inventory (TIPI-J). Japanese J Personal. 2012;21: 40–52. doi: 10.2132/personality.21.40

[22] Gosling SD, Rentfrow PJ, Swann WB. A very brief measure of the Big-Five personality domains. J Res Pers. 2003;37: 504–528. doi: 10.1016/S0092-6566(03)00046-1

[23] Iwasa H, Yoshida Y. Psychometric evaluation of the Japanese version of Ten Item Personality Inventory (TIPI-J) among middle-aged and elderly adults: Concurrent validity, internal consistency and test-retest reliability. Cogent Psychol. 2018;5. doi: 10.1080/23311908.2018.1426256

[24] Svensson S, Kjellgren KI, Ahlner J, Säljö R. Reasons for adherence with antihypertensive medication. Int J Cardiol. 2000;76: 157–163. doi: 10.1016/s0167-5273(00)00374-0 11104870

[25] Morisky DE, Ang A, Krousel-Wood M, Ward HJ. Predictive validity of a medication adherence measure in an outpatient setting. J Clin Hypertens. 2008;10: 348–354. doi: 10.1111/j.1751-7176.2008.07572.x 18453793 PMC2562622

[26] Ueno H, Yamazaki Y, Yonekura Y, Park MJ, Ishikawa H, Kiuchi T. Reliability and validity of a 12-item medication adherence scale for patients with chronic disease in Japan. BMC Health Serv Res. 2018;18: 1–9. doi: 10.1186/s12913-018-3380-7 30064422 PMC6069892

[27] Ueno H, Ishikawa H, Kato M, Okuhara T, Okada H, Kiuchi T. Factors related to self-care drug treatment and medication adherence of elderly people in Japan. Public Heal Pract. 2021;2: 100106. doi: 10.1016/j.puhip.2021.100106 36101625 PMC9461522

[28] Charlson ME, Pompei P, Ales KL, MacKenzie CR. A new method of classifying prognostic comorbidity in longitudinal studies: Development and validation. J Chronic Dis. 1987;40: 373–383. doi: 10.1016/0021-9681(87)90171-8 3558716

[29] Peduzzi P, Concato J, Kemper E, Holford TR, Feinstein AR. A simulation study of the number of events per variable in logistic regression analysis. J Clin Epidemiol. 1996;49: 1373–1379. doi: 10.1016/s0895-4356(96)00236-3 8970487

[30] Piña IL, Di Palo KE, Brown MT, Choudhry NK, Cvengros J, Whalen D, et al. Medication adherence: Importance, issues and policy: A policy statement from the American Heart Association. Prog Cardiovasc Dis. 2021;64: 111–120. doi: 10.1016/j.pcad.2020.08.003 32800791

[31] Simon ST, Kini V, Levy AE, Ho PM. Medication adherence in cardiovascular medicine. BMJ. 2021;374. doi: 10.1136/bmj.n1493 34380627

[32] Tabernero C, Gutiérrez-Domingo T, Vecchione M, Cuadrado E, Castillo-Mayén R, Rubio S, et al. A longitudinal study on perceived health in cardiovascular patients: The role of conscientiousness, subjective wellbeing and cardiac self-efficacy. PLoS One. 2019;14: 1–13. doi: 10.1371/journal.pone.0223862 31622377 PMC6797191

[33] Axelsson M, Brink E, Lundgren J, Lötvall J. The influence of personality traits on reported adherence to medication in individuals with chronic disease: An Epidemiological study in West Sweden. PLoS One. 2011;6: 1–7. doi: 10.1371/journal.pone.0018241 21464898 PMC3065484

[34] Hazrati-Meimaneh Z, Amini-Tehrani M, Pourabbasi A, Gharlipour Z, Rahimi F, Ranjbar-Shams P, et al. The impact of personality traits on medication adherence and self-care in patients with type 2 diabetes mellitus: The moderating role of gender and age. J Psychosom Res. 2020;136: 110178. doi: 10.1016/j.jpsychores.2020.110178 32623192

[35] Shiraishi Y, Kohsaka S, Sato N, Takano T, Kitai T, Yoshikawa T, et al. 9-year trend in the management of acute heart failure in Japan: A report from the national consortium of acute heart failure registries. J Am Heart Assoc. 2018;7. doi: 10.1161/JAHA.118.008687 30371201 PMC6222932

[36] Mora PA, Hahn E, Leventhal H, Ceric F. Elucidating the relationship between negative affectivity and symptoms: The role of illness-specific affective responses. Ann Behav Med. 2007;34: 77–86. doi: 10.1007/BF02879923 17688399

[37] Honda K, Jacobson JS. Use of complementary and alternative medicine among United States adults: The influences of personality, coping strategies, and social support. Prev Med (Baltim). 2005;40: 46–53. doi: 10.1016/j.ypmed.2004.05.001 15530580

[38] Jokela M, Batty GD, Nyberg ST, Virtanen M, Nabi H, Singh-Manoux A, et al. Personality and all-cause mortality: Individual-participant meta-analysis of 3,947 deaths in 76,150 adults. Am J Epidemiol. 2013;178: 667–675. doi: 10.1093/aje/kwt170 23911610 PMC3755650

[39] Hakulinen C, Hintsanen M, Munafò MR, Virtanen M, Kivimäki M, Batty GD, et al. Personality and smoking: Individual-participant meta-analysis of nine cohort studies. Addiction. 2015;110: 1844–1852. doi: 10.1111/add.13079 26227786 PMC4609271

