# Supplementary material for: Association between the Big Five personality traits and medication adherence in patients with cardiovascular disease: A cross-sectional study
Source: PLoS One. 2022 Dec 1;17(12):e0278534. doi: 10.1371/journal.pone.0278534 (PMC9714849; doi:10.1371/journal.pone.0278534)
Supplement: S1 Table — (DOCX) [file pone.0278534.s001.docx]

**S1 Table.** **Big Five personality traits according to the patient characteristics**

|  | Age | |  |
| --- | --- | --- | --- |
|  | < 70 years (n=64) | ≥ 70 years (n=64) | p* |
| Conscientiousness | 3.95 (1.04) | 4.24 (1.23) | 0.16 |
| Neuroticism | 4.26 (0.90) | 3.88 (1.08) | 0.034 |
| Openness | 3.85 (1.03) | 3.74 (1.13) | 0.57 |
| Extraversion | 4.07 (1.03) | 4.12 (1.20) | 0.84 |
| Agreeableness | 3.44 (0.98) | 2.97 (1.28) | 0.022 |
|  |  |  |  |
|  | Sex | |  |
|  | Women (n=34) | Men (n=94) | p* |
| Conscientiousness | 4.15 (1.43) | 4.08 (1.08) | 0.77 |
| Neuroticism | 3.96 (0.94) | 4.11 (1.03) | 0.44 |
| Openness | 3.88 (1.09) | 3.77 (1.08) | 0.59 |
| Extraversion | 3.96 (1.09) | 4.21 (1.08) | 0.65 |
| Agreeableness | 2.88 (0.96) | 3.31 (1.21) | 0.060 |
|  |  |  |  |
|  | Complication of heart failure | |  |
|  | No (n=79) | Yes (n=49) | p* |
| Conscientiousness | 4.02 (1.18) | 4.21 (1.09) | 0.37 |
| Neuroticism | 4.05 (0.98) | 4.09 (1.05) | 0.85 |
| Openness | 3.87 (1.10) | 3.67 (1.04) | 0.31 |
| Extraversion | 4.18 (1.24) | 3.94 (0.85) | 0.23 |
| Agreeableness | 3.42 (1.12) | 2.86 (1.15) | 0.007 |
|  |  |  |  |
|  | Number of prescribed medications | |  |
|  | <5 (n=41) | ≥ 5 (n=87) | p* |
| Conscientiousness | 4.21 (1.17) | 4.05 (1.14) | 0.46 |
| Neuroticism | 3.85 (1.00) | 4.17 (0.99) | 0.094 |
| Openness | 4.02 (1.11) | 3.69 (1.05) | 0.10 |
| Extraversion | 4.07 (1.04) | 4.10 (1.14) | 0.91 |
| Agreeableness | 3.24 (1.12) | 3.18 (1.19) | 0.79 |

The score of each personality trait is expressed by mean (standard deviation).

*p for Student’s t test
