# Supplementary material for: Association between the Big Five personality traits and medication adherence in patients with cardiovascular disease: A cross-sectional study
Source: PLoS One. 2022 Dec 1;17(12):e0278534. doi: 10.1371/journal.pone.0278534 (PMC9714849; doi:10.1371/journal.pone.0278534)
Supplement: S2 Table — (DOCX) [file pone.0278534.s002.docx]

**S2 Table.** **Results of multivariate logistic regression analysis for the association between Big Five personality traits and four domains of medication adherence (dependent variable)**

**Dependent variable: High compliance**

|  | OR | (95% CI) | p |
| --- | --- | --- | --- |
| Conscientiousness, per 1 pt | 1.90 | (1.30–2.79) | 0.001 |
| Age, per 1yr | 1.00 | (0.96–1.03) | 0.819 |
| Sex (women) | 0.79 | (0.31–2.01) | 0.621 |
| Complication of heart failure | 1.01 | (0.38–2.66) | 0.983 |
| Number of prescribed medications, per 1 drug | 0.93 | (0.74–1.17) | 0.549 |
| Charlson Comorbidity Index, per 1 pt | 1.25 | (0.76–2.07) | 0.380 |
| Neuroticism, per 1 pt | 0.98 | (0.68– 1.42) | 0.931 |
| Age, per 1yr | 1.01 | (0.97–1.04) | 0.739 |
| Sex (women) | 0.75 | (0.31–1.81) | 0.527 |
| Complication of heart failure | 1.11 | (0.44–2.78) | 0.825 |
| Number of prescribed medications, per 1 drug | 0.88 | (0.71–1.11) | 0.260 |
| Charlson Comorbidity Index, per 1 pt | 1.27 | (0.78–2.06) | 0.335 |
| Extraversion, per 1 pt | 0.95 | (0.69–1.31) | 0.760 |
| Age, per 1yr | 1.00 | (0.98–1.04) | 0.708 |
| Sex (women) | 0.75 | (0.31–1.80) | 0.517 |
| Complication of heart failure | 1.10 | (0.44–2.77) | 0.837 |
| Number of prescribed medications, per 1 drug | 0.88 | (0.71–1.10) | 0.269 |
| Charlson Comorbidity Index, per 1 pt | 1.26 | (0.77–2.05) | 0.352 |
| Openness, per 1 pt | 1.14 | (0.82–1.61) | 0.428 |
| Age, per 1yr | 1.01 | (0.98–1.04) | 0.685 |
| Sex (women) | 0.74 | (0.31–1.79) | 0.505 |
| Complication of heart failure | 1.12 | (0.44–2.81) | 0.815 |
| Number of prescribed medications, per 1 drug | 0.89 | (0.71–1.11) | 0.299 |
| Charlson Comorbidity Index, per 1 pt | 1.28 | (0.78–2.10) | 0.323 |
| Agreeableness, per 1 pt | 1.01 | (0.73–1.41) | 0.936 |
| Age, per 1yr | 1.00 | (0.98–1.04) | 0.708 |
| Sex (women) | 0.76 | (0.31–1.82) | 0.531 |
| Complication of heart failure | 1.12 | (0.44–2.83) | 0.819 |
| Number of prescribed medications, per 1 drug | 0.88 | (0.71–1.11) | 0.258 |
| Charlson Comorbidity Index, per 1 pt | 1.27 | (0.78–2.06) | 0.336 |

**Dependent variable: High collaboration**

|  | OR | (95% CI) | p |
| --- | --- | --- | --- |
| Conscientiousness, per 1 pt | 1.90 | (1.31–2.76) | 0.001 |
| Age, per 1yr | 0.97 | (0.94–1.01) | 0.110 |
| Sex (women) | 0.78 | (0.31–1.96) | 0.603 |
| Complication of heart failure | 0.83 | (0.31–2.20) | 0.701 |
| Number of prescribed medications, per 1 drug | 1.21 | (0.97–1.53) | 0.091 |
| Charlson Comorbidity Index, per 1 pt | 0.94 | (0.58–1.54) | 0.817 |
| Neuroticism, per 1 pt | 1.29 | (0.88–1.87) | 0.190 |
| Age, per 1yr | 1.00 | (0.96–1.02) | 0.551 |
| Sex (women) | 0.74 | (0.31–1.77) | 0.502 |
| Complication of heart failure | 0.92 | (0.37–2.31) | 0.867 |
| Number of prescribed medications, per 1 drug | 1.11 | (0.90–1.38) | 0.334 |
| Charlson Comorbidity Index, per 1 pt | 0.97 | (0.61–1.55) | 0.898 |
| Extraversion, per 1 pt | 1.34 | (0.96–1.88) | 0.083 |
| Age, per 1yr | 0.98 | (0.96–1.01) | 0.317 |
| Sex (women) | 0.78 | (0.32–1.86) | 0.569 |
| Complication of heart failure | 0.97 | (0.38–2.45) | 0.950 |
| Number of prescribed medications, per 1 drug | 1.11 | (0.89–1.37) | 0.364 |
| Charlson Comorbidity Index, per 1 pt | 1.01 | (0.63–1.63) | 0.958 |
| Openness, per 1 pt | 0.91 | (0.66–1.30) | 0.600 |
| Age, per 1yr | 0.99 | (0.96–1.01) | 0.334 |
| Sex (women) | 0.76 | (0.32–1.82) | 0.541 |
| Complication of heart failure | 0.93 | (0.37–2.32) | 0.878 |
| Number of prescribed medications, per 1 drug | 1.11 | (0.90–1.38) | 0.318 |
| Charlson Comorbidity Index, per 1 pt | 0.97 | (0.61–1.55) | 0.897 |
| Agreeableness, per 1 pt | 1.20 | (0.87–1.66) | 0.274 |
| Age, per 1yr | 0.99 | (0.96–1.02) | 0.486 |
| Sex (women) | 0.78 | (0.33–1.87) | 0.580 |
| Complication of heart failure | 1.02 | (0.40–2.59) | 0.963 |
| Number of prescribed medications, per 1 drug | 1.13 | (0.91–1.40) | 0.258 |
| Charlson Comorbidity Index, per 1 pt | 0.97 | (0.30–1.55) | 0.889 |

**Dependent variable: High willingness**

|  | OR | (95% CI) | p |
| --- | --- | --- | --- |
| Conscientiousness, per 1 pt | 1.74 | (1.19–2.54) | 0.004 |
| Age, per 1yr | 0.95 | (0.92–0.98) | 0.004 |
| Sex (women) | 0.95 | (0.38–2.36) | 0.904 |
| Complication of heart failure | 1.00 | (0.36–2.76) | 0.994 |
| Number of prescribed medications, per 1 drug | 1.12 | (0.89–1.42) | 0.345 |
| Charlson Comorbidity Index, per 1 pt | 0.91 | (0.55–1.51) | 0.718 |
| Neuroticism, per 1 pt | 1.45 | (0.97–2.18) | 0.072 |
| Age, per 1yr | 0.97 | (0.93–1.00) | 0.053 |
| Sex (women) | 0.90 | (0.36–2.21) | 0.813 |
| Complication of heart failure | 1.04 | (0.39–2.76) | 0.943 |
| Number of prescribed medications, per 1 drug | 1.03 | (0.83–1.29) | 0.768 |
| Charlson Comorbidity Index, per 1 pt | 0.94 | (0.58–1.54) | 0.819 |
| Extraversion, per 1 pt | 0.96 | (0.69–1.34) | 0.815 |
| Age, per 1yr | 0.96 | (0.93–0.99) | 0.017 |
| Sex (women) | 0.93 | (0.38–2.26) | 0.867 |
| Complication of heart failure | 1.05 | (0.40–2.76) | 0.926 |
| Number of prescribed medications, per 1 drug | 1.05 | (0.84–1.32) | 0.643 |
| Charlson Comorbidity Index, per 1 pt | 0.95 | (0.58–1.55) | 0.841 |
| Openness, per 1 pt | 1.07 | (0.76–1.52) | 0.683 |
| Age, per 1yr | 0.96 | (0.93–0.99) | 0.018 |
| Sex (women) | 0.92 | (0.38–2.26) | 0.859 |
| Complication of heart failure | 1.06 | (0.40–2.79) | 0.909 |
| Number of prescribed medications, per 1 drug | 1.06 | (0.85–1.32) | 0.324 |
| Charlson Comorbidity Index, per 1 pt | 0.96 | (0.59–1.56) | 0.869 |
| Agreeableness, per 1 pt | 1.19 | (0.85–1.68) | 0.309 |
| Age, per 1yr | 0.96 | (0.93–1.00) | 0.031 |
| Sex (women) | 0.96 | (0.39–2.36) | 0.928 |
| Complication of heart failure | 1.14 | (0.43–3.06) | 0.794 |
| Number of prescribed medications, per 1 drug | 1.06 | (0.85–1.32) | 0.613 |
| Charlson Comorbidity Index, per 1 pt | 0.95 | (0.58–1.55) | 0.832 |

**Dependent variable: High acceptance**

|  | OR | (95% CI) | p |
| --- | --- | --- | --- |
| Conscientiousness, per 1 pt | 1.31 | (0.86–1.85) | 0.236 |
| Age, per 1yr | 0.99 | (0.96–1.03) | 0.748 |
| Sex (women) | 0.59 | (0.23–1.37) | 0.203 |
| Complication of heart failure | 2.19 | (0.86–5.90) | 0.100 |
| Number of prescribed medications, per 1 drug | 1.13 | (0.87–1.35) | 0.473 |
| Charlson Comorbidity Index, per 1 pt | 0.82 | (0.51–1.34) | 0.446 |
| Neuroticism, per 1 pt | 1.23 | (0.88–1.72) | 0.225 |
| Age, per 1yr | 0.99 | (0.96–1.02) | 0.509 |
| Sex (women) | 0.58 | (0.24–1.41) | 0.232 |
| Complication of heart failure | 2.34 | (0.88–6.17) | 0.087 |
| Number of prescribed medications, per 1 drug | 1.08 | (0.87–1.35) | 0.479 |
| Charlson Comorbidity Index, per 1 pt | 0.85 | (0.52–1.39) | 0.521 |
| Extraversion, per 1 pt | 1.23 | (0.88–1.72) | 0.225 |
| Age, per 1yr | 0.99 | (0.96–1.02) | 0.509 |
| Sex (women) | 0.58 | (0.24–1.41) | 0.232 |
| Complication of heart failure | 2.34 | (0.88–6.17) | 0.087 |
| Number of prescribed medications, per 1 drug | 1.08 | (0.87–1.35) | 0.479 |
| Charlson Comorbidity Index, per 1 pt | 0.85 | (0.52–1.39) | 0.521 |
| Openness, per 1 pt | 0.89 | (0.64–1.25) | 0.518 |
| Age, per 1yr | 0.99 | (0.96–1.02) | 0.510 |
| Sex (women) | 0.58 | (0.24–1.41) | 0.228 |
| Complication of heart failure | 2.24 | (0.86–5.88) | 0.100 |
| Number of prescribed medications, per 1 drug | 1.09 | (0.87–1.35) | 0.466 |
| Charlson Comorbidity Index, per 1 pt | 0.83 | (0.51–1.34) | 0.441 |
| Agreeableness, per 1 pt | 0.91 | (0.65–1.27) | 0.584 |
| Age, per 1yr | 0.99 | (0.96–1.02) | 0.467 |
| Sex (women) | 0.56 | (0.23–1.37) | 0.203 |
| Complication of heart failure | 2.16 | (0.82–5.71) | 0.120 |
| Number of prescribed medications, per 1 drug | 1.09 | (0.88–1.36) | 0.436 |
| Charlson Comorbidity Index, per 1 pt | 0.84 | (0.52–1.35) | 0.462 |

OR, odds ratio; CI, confidence interval

Complication of heart failure is defined as heart failure hospitalization and/or prior heart failure.
